# Supplementary material for: Fecal microbiota transplantation in HIV: A pilot placebo-controlled study
Source: Nat Commun. 2021 Feb 18;12:1139. doi: 10.1038/s41467-021-21472-1 (PMC7892558; doi:10.1038/s41467-021-21472-1)
Supplement: Supplementary file 1 — Supplementary Information [file 41467_2021_21472_MOESM1_ESM.pdf]

Fecal microbiota transplantation in HIV: A pilot placebo-controlled study

Sergio Serrano-Villar, Alba Talavera-Rodríguez, María José Gosalbes, Nadia Madrid, José Antonio Pérez-Molina, Ryan J. Elliott, Beatriz Navia, Val F. Lanza, Alejandro Vallejo, Majdi Osman, Fernando Dronda, Shrish Budree, Javier Zamora, Carolina Gutiérrez, Mónica Manzano, María Jesús Vivancos, Raquel Ron, Javier Martínez-Sanz, Sabina Herrera, Uxua Ansa, Andrés Moya, Santiago Moreno

– Supplementary Information –

This file contains:

1.Supplementary tables

2. Supplementary figure legends

Supplementary tables

Supplementary Table 1. Individual metadata

id	Group	Donor	Age	Sex	AIDS events	Nadir CD4 T cell	CD4+ T cells	CD4/CD8 ratio	Antibiotics in the past 6 months	Antibiotics in the past 3 months	Completed Follow-up
R1	FMT	485	53	Male	Yes	15	532	0,45	Yes	Yes	Yes
R2	FMT	485	48	Female	No	80	1747	0,59	Yes	Yes	Yes
R3	Placebo	485	54	Male	Yes	170	478	0,38	No	No	Yes
R4	FMT	485	37	Male	No	465	715	0,53	No	No	Yes
R5	Placebo	485	53	Male	Yes	110	533	0,40	No	No	Yes
R6	Placebo	485	28	Male	No	287	330	0,20	Yes	Yes	Yes
R7	FMT	543	53	Male	Yes	26	847	0,37	No	No	Yes
R8	Placebo	543	48	Male	No	284	684	1,02	Yes	Yes	Yes
R9	Placebo	543	59	Male	No	258	410	0,25	No	No	Yes
R10	Placebo	543	34	Male	No	378	556	0,96	No	No	Yes
R11	FMT	543	54	Male	Yes	30	821	0,91	No	No	Yes
R12	FMT	543	42	Male	No	180	966	0,73	No	No	Yes
R13	Placebo	505	41	Female	Yes	100	832	0,89	No	No	Yes
R14	FMT	505	58	Male	No	70	692	0,68	No	Yes	Yes
R15	Placebo	505	60	Male	No	160	521	0,85	No	No	Yes
R16	Placebo	505	43	Male	No	110	704	0,52	No	No	Yes
R17	FMT	505	59	Male	No	113	853	0,87	No	No	Yes
R18	FMT	505	51	Male	No	56	326	0,18	No	No	Yes
R19	Placebo	505	36	Male	No	274	400	0,48	No	No	Yes
R20	FMT	543	25	Male	No	250	465	0,83	No	No	Yes
R21	Placebo	543	49	Transgender	No	300	454	0,96	Yes	Yes	Yes
R22	Placebo	505	31	Male	No	249	609	0,99	No	No	Yes
R23	FMT	505	59	Female	No	187	457	0,31	No	No	Yes
R24	FMT	505	56	Male	No	106	961	0,79	No	No	Yes
R25	Placebo	543	33	Male	No	400	609	0,72	No	No	Yes
R26	Placebo	485	46	Male	No	259	685	0,74	Yes	No	Yes
R27	Placebo	485	69	Male	No	356	461	0,73	No	No	Yes
R28	FMT	543	30	Male	No		535	0,56	No	No	Yes
R29	FMT	485	43	Male	Yes	126	346	0,48	Yes	Yes	No
R30	FMT	485	41	Male	No	321	416	0,58	No	No	Yes

Some potential patient identifiers are not displayed to avoid compromising patient's anonimity

Supplementary Table 2. Timing of antibiotic use (in weeks) with respect to the baseline (pre-intervention) time point.

		Week														Antibiotic prescribed and reason
ID	Donor	Pre-Study	W0	W1	W2	W3	W4	W5	W6	W7	W8	W12	W24	W36	W48	
1	A	-14														Amoxicillin/clavulanate 10days; upper respiratory infection.
2	A	-0.2														Amoxicillin/clavulanate 7days; upper respiratory infection
3	Placebo															
4	A															
5	Placebo															
6	Placebo	-4														Amoxicillin, Chlarythromycin, Omeprazol 20 days; H. pylori infection
7	C															
8	Placebo	-2.8														Ceftriaxone + Azithromycin (single doses); Urethritis
9	Placebo															
10	Placebo															
11	C															
12	C															
13	Placebo															
14	B			1.3												Azithromycin 5 days; Upper respiratory infection.
15	Placebo															
16	Placebo															
17	B															
18	B											23.5				Fluconazol, 15 days; oral candidiasis
18	B												26.7			Nitazoxanide, 15 days; cryptosporidiasis
18	B													44.3		Penicillin G 2.4 MUI 3 doses; latent syphilis
19	Placebo					3.6										Amoxicillin/clavulanate 4 days; back pain
20	C															
21	Placebo	-10														Amoxicillin/clavulanate 5 days; odontogenic infection
22	Placebo															
23	B												33			Azithromycin 3days; upper respiratory infection
24	B												33			Amoxicillin 7 days; upper respiratory infection
25	Placebo												33			Fosfomycin 3 days; urinary tract infection
26	Placebo													41		Cefalexine 1 day; prophylaxis for spine surgery
27	Placebo													46		Amoxicillin/clavulanate 2days; upper respiratory infection
29	A	-5.8														Amoxicillin/clavulanate 5 days; odontogenic infection
30	A										10.6					Amoxicillin/clavulanate 5 days; pharyngoamigdalitis

Subjects in the FMT arm with recent antibiotic exposure before the first FMT highlighted in blue, during the FMT procedures highlighted in green. In blue: individuals in the FMT group who received antibiotics before the first FMT; in green, individuals in the FMT group who received antibiotics during FMT treatment; in orange, individuals who received antibiotics in the FMT group after the treatment period. In red

Abbreviatures: FMT, fecal microbiota transplant.

Supplementary Table 3. Food consumption and energy and nutrient intake in each group

	FMT	Placebo	P value
**Cereals and pulses (g/d)**	259.6 (73.2)	219.1 (121.4)	0.077
**Greens and vegetables (g/d)**	352.7 (185.1)	319.8 (182.0)	0.682
**Fruits (g/d)**	325.6 (291.7)	342.2 (256.3)	0.959
**Milk products (g/d)**	268.8 (165.7)	297.5 (188.7)	0.644
**Meat and meat products (g/d)**	133.6 (88.9)	194.0 (141.2)	0.238
**Fish and fish products (g/d)**	88.6 (55.6)	90.1 (108.3)	0.397
**Eggs (g/d)**	22.7 (38.3)	28.4 (40.5)	0.850
**Sugars, sweets, pastries (g/d)**	34.8 (60.7)	7.7 (9.2)	0.255
**Fats and oils (g/d)**	30.7 (16.7)	33.0 (14.0)	0.573
**Drinks (g/d)**	1220.9 (520.3)	1072.9 (844.2)	0.191
**Prepared and pre-cooked food (g/d)**	12.1 (27.9)	6.5 (22.1)	0.083
**Aperitifs (g/d)**	9.5 (13.3)	18.9 (44.8)	0.722
**Condiments and sauces (g/d)**	13.3 (10.2)	8.0 (10.7)	0.077
**Others (g/d)**	0.0 (0.1)	0.9 (3.3)	0.956
**Energy (kcal/d)**	2380.5 (390)	2409 (569)	0.762
**Proteins (g/d)**	95.6 (19.8)	107.8 (41.3)	0.511
**Proteins (% TE)**	16.4 (3.1)	17.7 (4.3)	0.362
**Carbohydrates (g/d)**	239.0 (51.1)	244.1 (102.1)	0.511
**Carbohydrates (% TE)**	43.1 (7.0)	42.6 (10.7)	0.960
**Added sugars (% TE)**	15.8 (5.4)	15.5 (6.0)	0.960
**Total Fibers (g/d)**	31.9 (11.1)	24.6 (8.9)	0.072
**Total lipids (g/d)**	101.1 (29.6)	99.8 (31.5)	0.840
**Total lipids (% TE)**	37.7 (6.7)	37.3 (8.2)	0.960
**SFA (% TE)**	11.4 (3.6)	11.1 (2.9)	0.801
**PUFA (% TE)**	5.6 (2.4)	5.0 (1.3)	0.545
**MUFA (% TE)**	17.2 (3.2)	17.6 (4.9)	0.614
**Omega 3 (% TE)**	0.8 (0.5)	0.7 (0.4)	0.311
**Omega 6 (% TE)**	4.6 (2.3)	4.1 (1.0)	0.880
**Trans fatty acids (% TE)**	0.3 (0.1)	0.4 (0.2)	0.264
**Alpha linolenic acid (% TE)**	0.5 (0.2)	0.4 (0.2)	0.264
**Alcohol (g/d)**	9.3 (13.0)	7.6 (14.4)	0.479
**Healthy Eating Index (HEI)**	66.5 (17.7)	66.2 (13.1)	0.920

All values are expressed as mean (SD)
Drinks (water included); Aperitifs (olives, pork scratchings, crisps, etc.); Others (e.g., dietetic products)
TE: Total energy intake; SFA: Saturated fat; PUFA: Poliunsaturated fat; MUFA: Monounsaturated fat

Supplementary Table 4. Primers used for 16SrRNA gene amplification

**Full-length primer sequences (standard IUPAC nucleotide nomenclature)**	
**16S Forward Primer**	5’TCGTCGGCAGCGTCAGATGTGTATAAGAGACAGCCTACGGG NGGCWGCAG
**16S Reverse Primer**	5’GTCTCGTGGGCTCGGAGATGTGTATAAGAGACAGGACTACH VGGGTATCTAATCC

Klindworth A, Pruesse E, Schweer T, et al. Evaluation of general 16S ribosomal RNA gene PCR primers for classical and next-generation sequencing-based diversity studies. Nucleic Acids Res 2013;41.

Figures

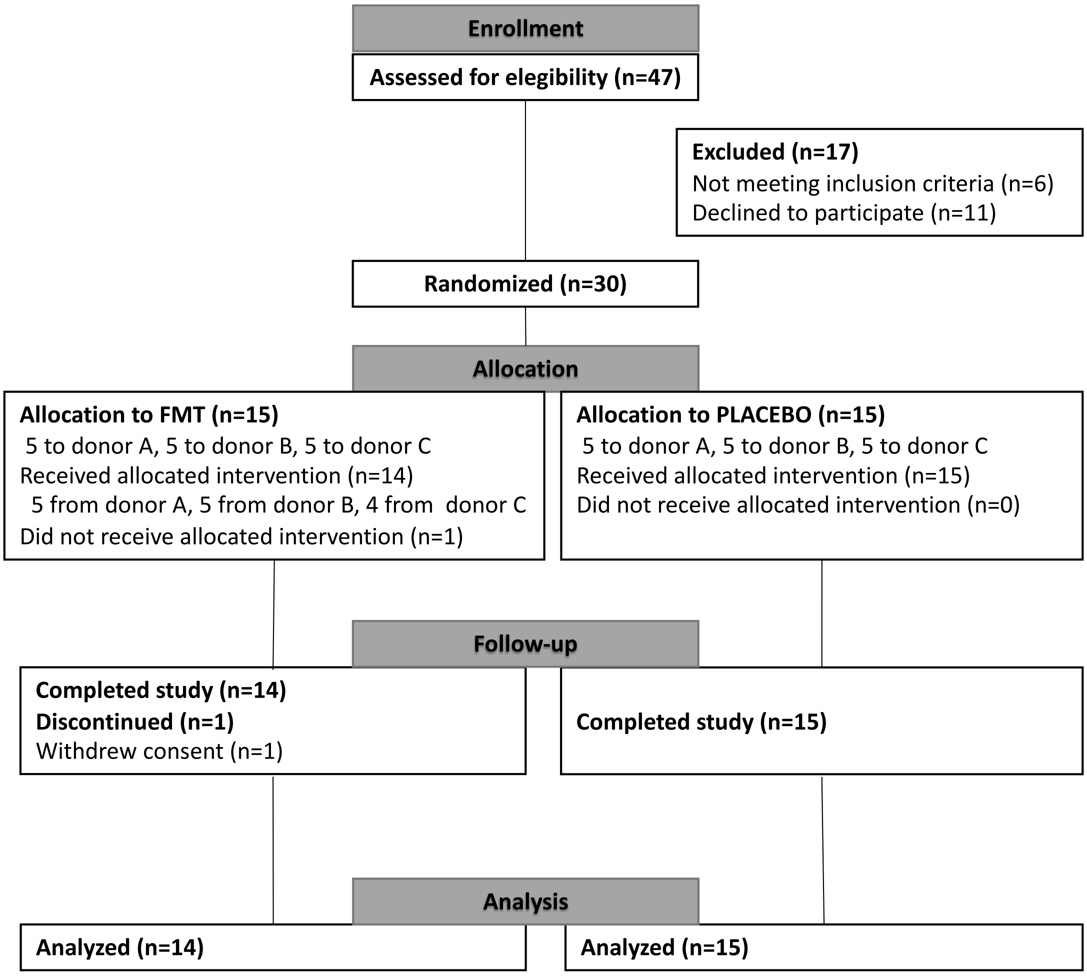

Supplementary Figure 1. Participant disposition.

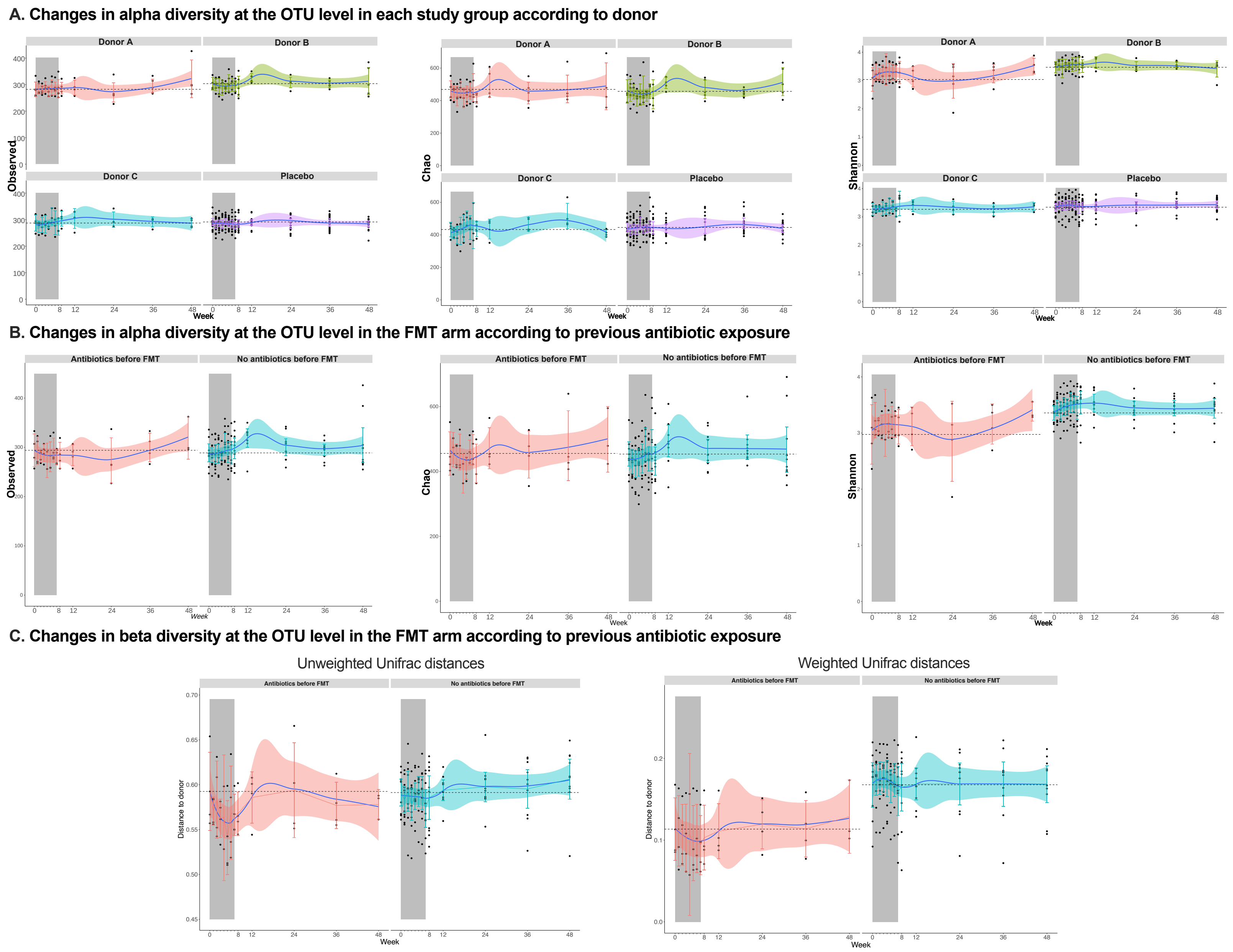

Supplementary Figure 2A. Changes in three metrics of alpha diversity at the OTU level in each study group according to donor. Figure S2B. Changes in three metrics of alpha diversity at the OTU level in the FMT arm according to antibiotic exposure before FMT (only subjects in the FMT are represented). Figure S2C. Engraftment of donor's microbiota on study participants. Weighted Unifrac distances from recipients to donor segregated by recent antibiotic exposure in the 14 subjects who received FMT. Horizontal dashed lines represent the baseline levels. Black dots represent individual measurements. Horizontal dashed lines represent the baseline levels. Blue lines represent the smoothed mean value. Vertical bars represent the 95% confidence intervals. The grey area indicates the induction period in which study participants received FMT or placebo. Two-sided P values estimated using mixed models not adjusted for multiple comparisons were estimated using mixed models. n=361 biologically independent samples from 14 individuals in the FMT group and 15 individuals in the placebo group.

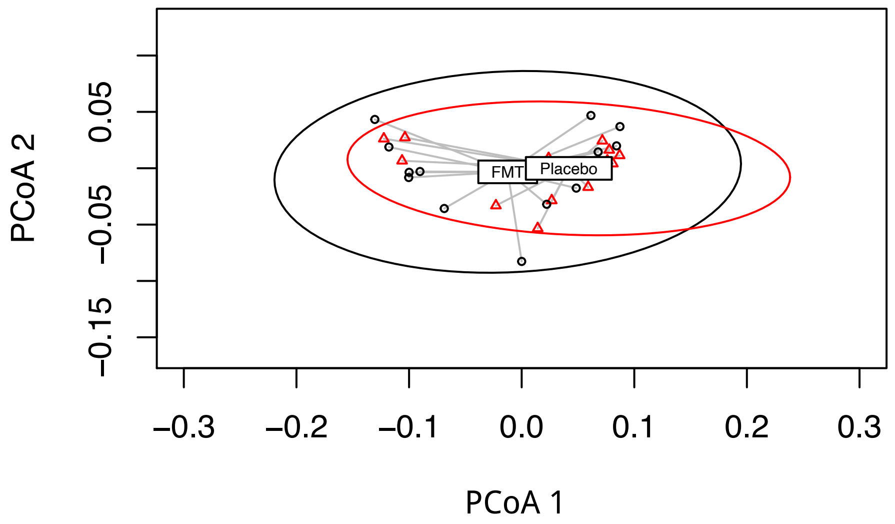

Supplementary Figure 3. Principal Coordinates Analysis (PCoA) of beta diversity distances (Genus level) between the FMT and placebo groups at baseline. ANOVA for the between-group mean distances, Adonis test, P value = 0.442.

n=29 biologically independent samples from 14 individuals in the FMT group and 15 individuals in the placebo group.

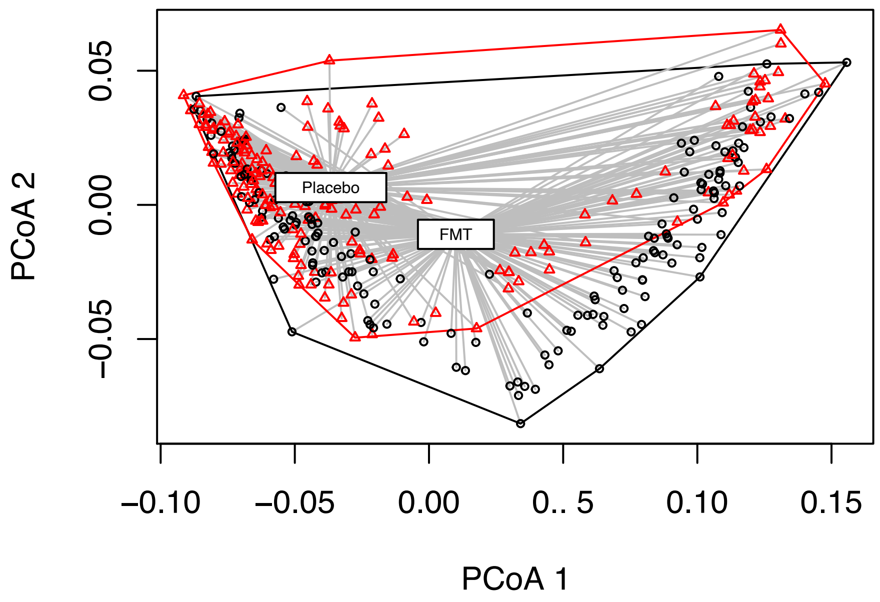

Supplementary Figure 4. PCoA of beta diversity distances (Genus level) in study samples within and between the FMT and placebo groups. ANOVA test, P = 0.0022; permuted P value = 0.01. n=361 biologically independent samples from 14 individuals in the FMT group and 15 individuals in the placebo group.

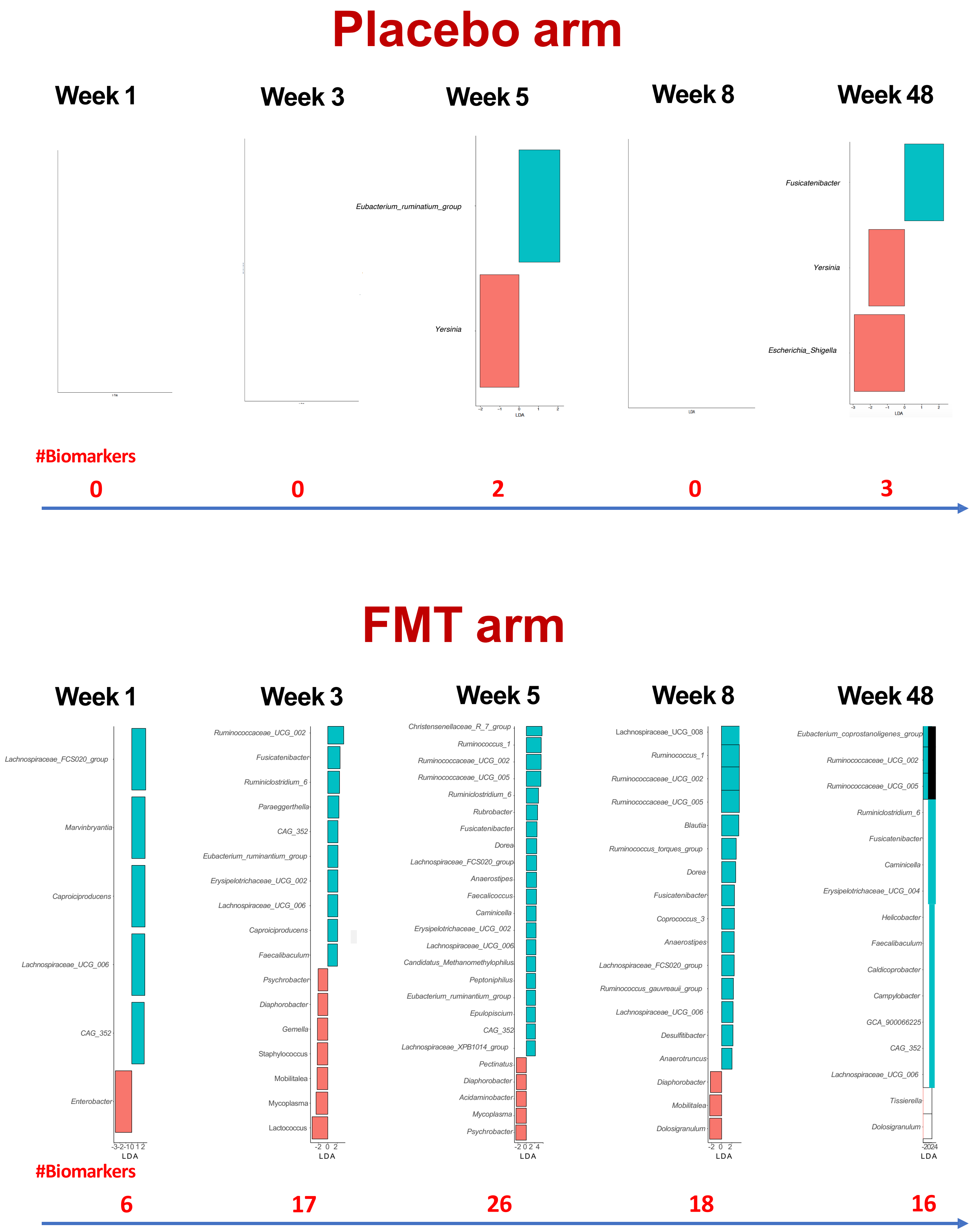

Supplementary Figure 5. LEfSe of LDA distances from baseline to different timepoints in each group. Red color indicates that the genus was depleted with respect to baseline, blue color indicates that the genus was enriched with respect to baseline. n=361 biologically independent samples from 14 individuals in the FMT group and 15 individuals in the placebo group.

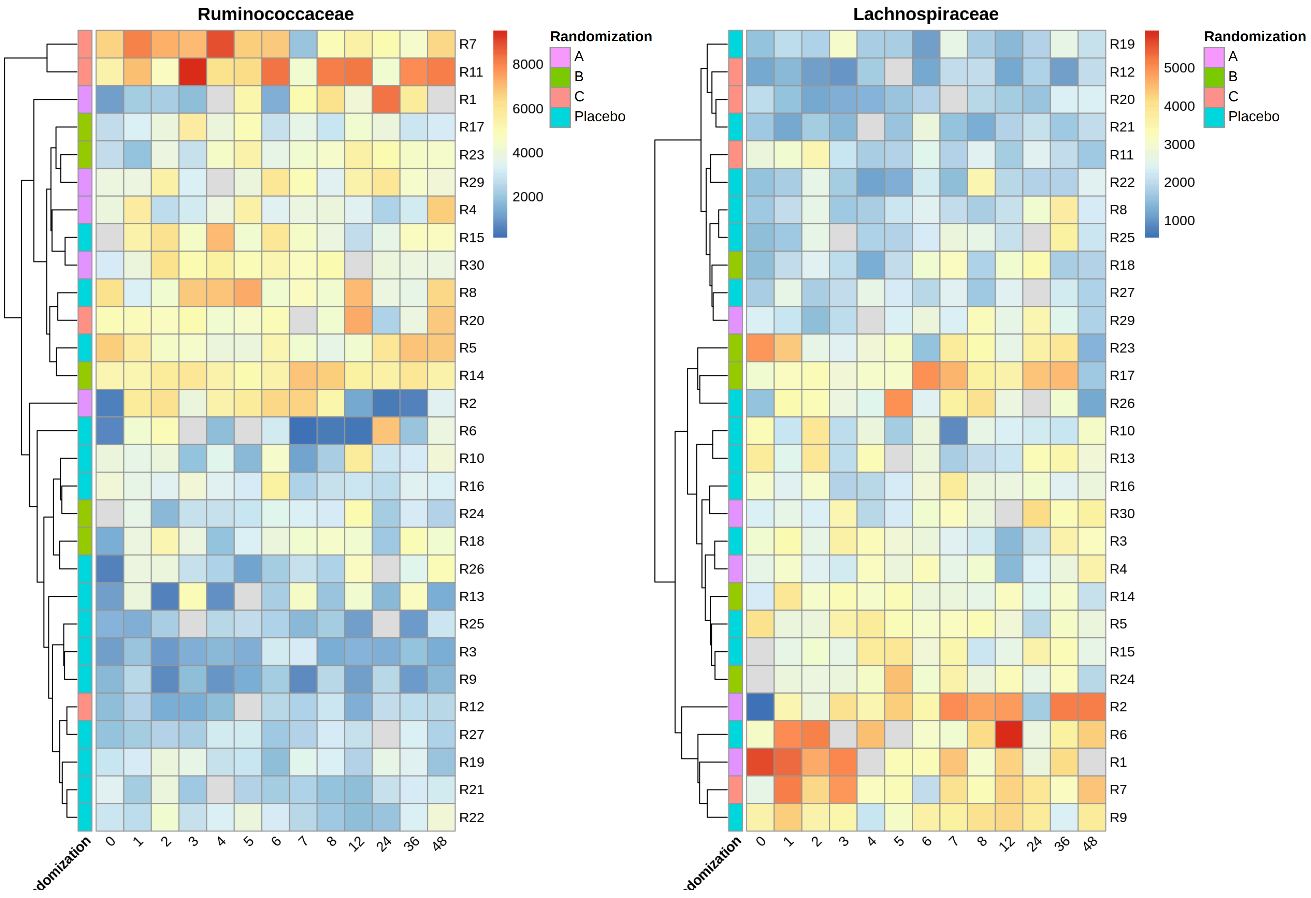

Supplementary Figure 6. Heatmap of Ruminococcaceae family and Lachnospiraceae family abundances (number of reads) during the study according to donor and study group. Hierarchical clustering was performed by rows.

n=369 biologically independent samples from 14 individuals in the FMT group, 15 individuals in the placebo group, and 3 donors.

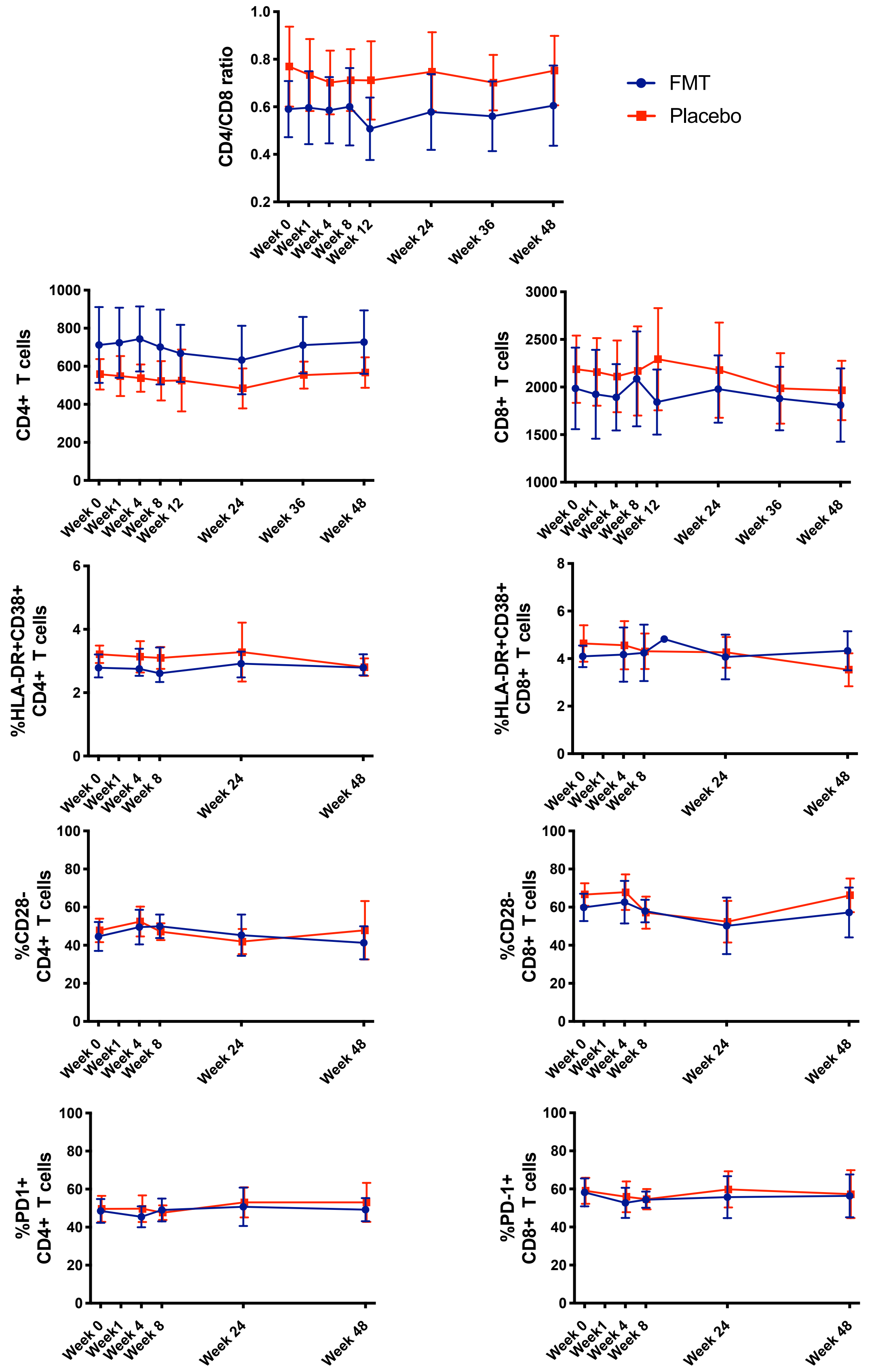

Supplementary Figure 7. Changes in circulating CD4+ T cells, CD8+ T cells, CD4/CD8 ratio, markers of T cell activation, senescence and exhaustion during the study. Lines represent mean values. Vertical bars represent the 95% confidence intervals. Two-sided P values estimated using mixed models not adjusted for multiple comparisons showed no significant differences between treatment arms.

n=120 biologically independent samples from 14 individuals in the FMT group, 15 individuals in the placebo group, and 3 donors.

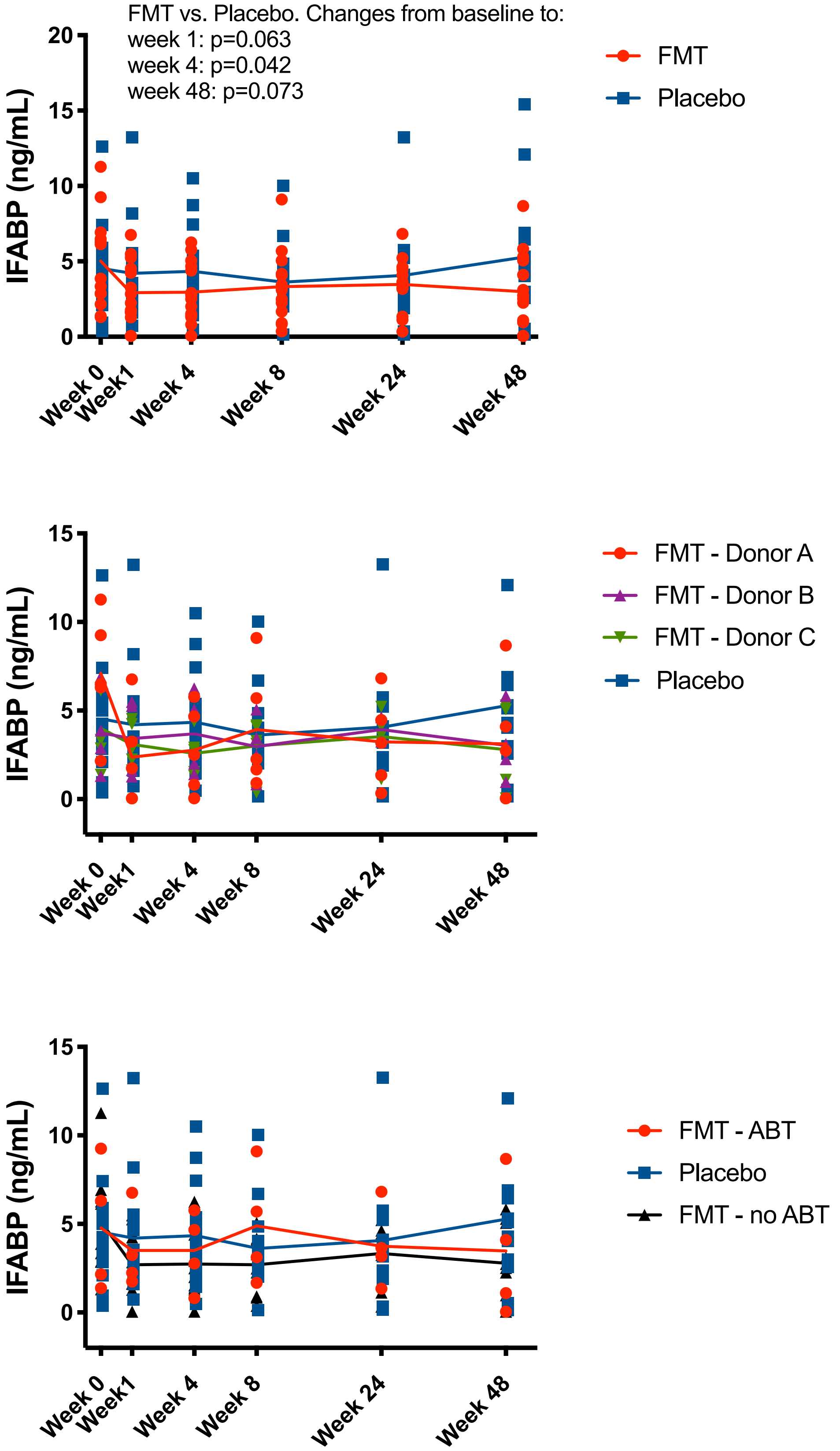

Supplementary Figure 8. Changes in plasma levels of IFABP. Each dot, square and triangle represent the IFABP mean value of an individual measurement (experiments run in triplicate). Lines represent group mean values. Vertical bars represent the 95% confidence intervals. Two-sided P values estimated using mixed models not adjusted for multiple comparisons are reported only for the grouped FMT vs. placebo comparison.

n=172 biologically independent samples from 14 individuals in the FMT group and 15 individuals in the placebo group.

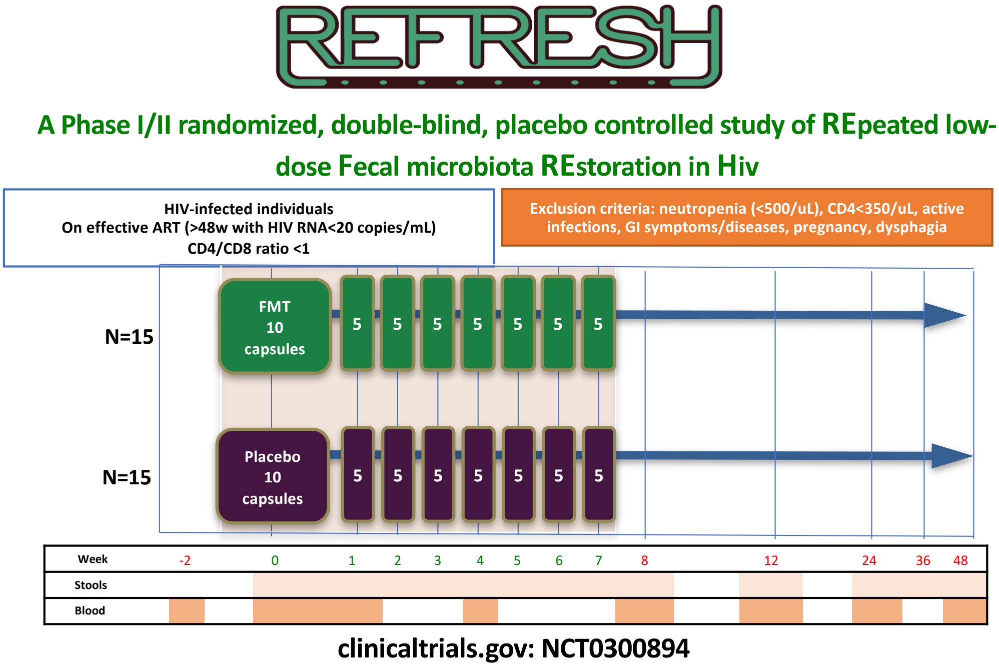

Supplementary Figure 9. Scheme of study design.

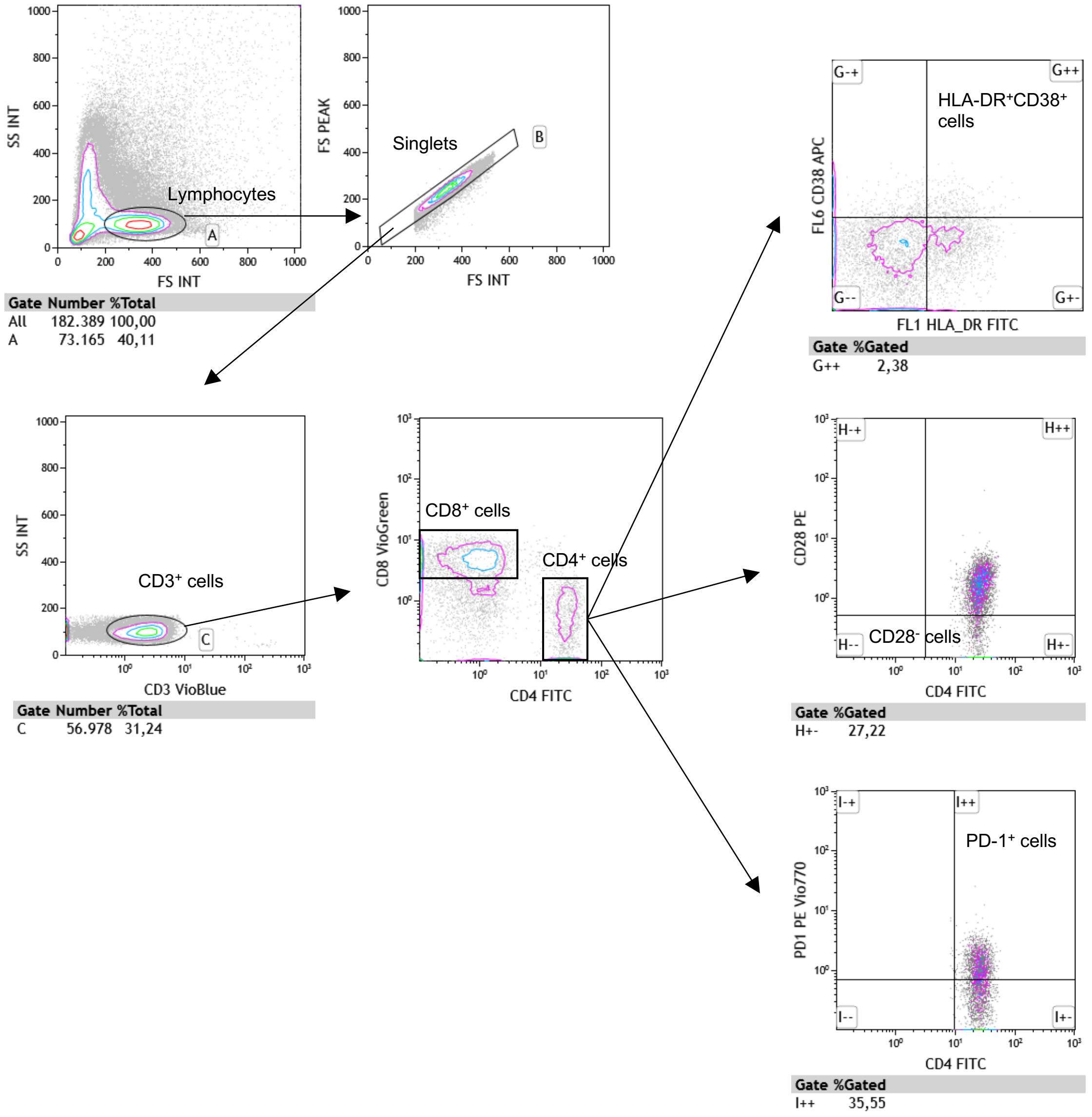

Supplementary Figure 10. Representative sample for flow-cytometric gating strategy. After initially gating lymphocytes according to morphological parameters only singlets were further analyzed. Then, cells with CD3+ phenotype were analyzed using CD4 and CD8 antibodies. Activated T cells were defined as the coexpression of CD38 and HLA-DR antibodies. Also, $CD28^-$ cells and $PD\text{-}1^+$ cells were analysed in the CD4+ or CD8+ cell subpopulation.
