# Supplementary material for: Fecal microbiota transplantation in HIV: A pilot placebo-controlled study
Source: Nat Commun. 2021 Feb 18;12:1139. doi: 10.1038/s41467-021-21472-1 (PMC7892558; doi:10.1038/s41467-021-21472-1)
Supplement: Supplementary file 3 — Description of Additional Supplementary Files [file 41467_2021_21472_MOESM3_ESM.docx]

**File Name: Supplementary Video 1**

**Description:** Principal Coordinates Analysis (PCoA) representing donor microbiota profiles and HIV-infected recipient microbiota community dynamics after the FMT or placebo intervention was generated using the Unweighted Unifrac distance metric at the OTU level. After FMT, recipient microbiota profiles remain distinct from the donors, but more pronounced changes are observed in the FMT group.
