# Supplementary figures and images for: Fecal microbiota transplantation in HIV: A pilot placebo-controlled study
Source: Nat Commun. 2021 Feb 18;12:1139. doi: 10.1038/s41467-021-21472-1 (PMC7892558; doi:10.1038/s41467-021-21472-1)

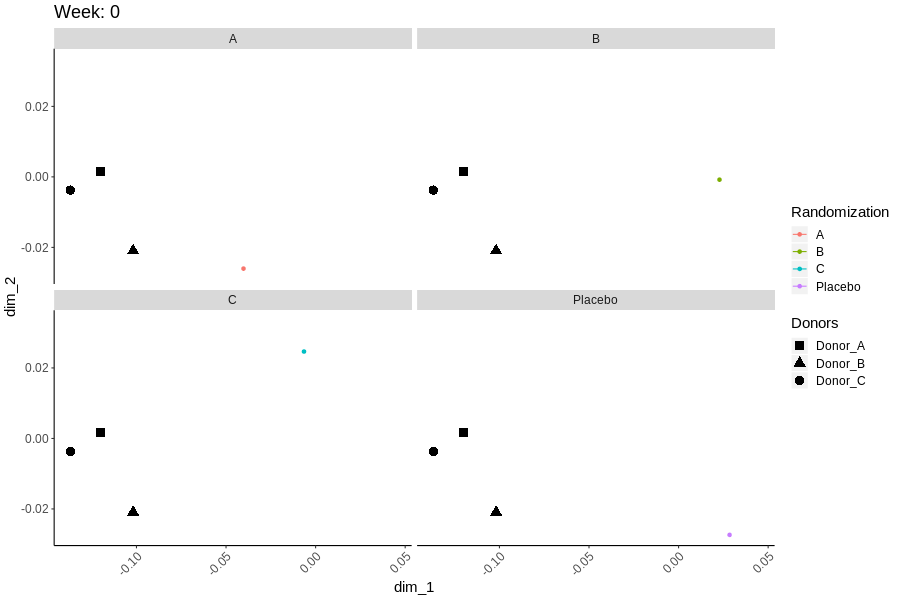

Supplement: Supplementary file 4 — Supplementary Video 1 [file 41467_2021_21472_MOESM4_ESM.gif]
