# Supplementary material for: Fecal microbiota transplantation in HIV: A pilot placebo-controlled study
Source: Nat Commun. 2021 Feb 18;12:1139. doi: 10.1038/s41467-021-21472-1 (PMC7892558; doi:10.1038/s41467-021-21472-1)
Supplement: Supplementary file 5 — Reporting Summary [file 41467_2021_21472_MOESM5_ESM.pdf]

nature research

Corresponding author(s): Sergio Serrano-Villar

Last updated by author(s): Jan 18, 2021

Reporting Summary

Nature Research wishes to improve the reproducibility of the work that we publish. This form provides structure for consistency and transparency in reporting. For further information on Nature Research policies, see our Editorial Policies and the Editorial Policy Checklist.

Statistics

For all statistical analyses, confirm that the following items are present in the figure legend, table legend, main text, or Methods section.

n/a	Confirmed	
☐	☒	The exact sample size (n) for each experimental group/condition, given as a discrete number and unit of measurement
☐	☒	A statement on whether measurements were taken from distinct samples or whether the same sample was measured repeatedly
☐	☒	The statistical test(s) used AND whether they are one- or two-sided<br>*Only common tests should be described solely by name; describe more complex techniques in the Methods section.*
☐	☒	A description of all covariates tested
☐	☒	A description of any assumptions or corrections, such as tests of normality and adjustment for multiple comparisons
☐	☒	A full description of the statistical parameters including central tendency (e.g. means) or other basic estimates (e.g. regression coefficient) AND variation (e.g. standard deviation) or associated estimates of uncertainty (e.g. confidence intervals)
☐	☒	For null hypothesis testing, the test statistic (e.g. F, t, r) with confidence intervals, effect sizes, degrees of freedom and P value noted<br>*Give P values as exact values whenever suitable.*
☒	☐	For Bayesian analysis, information on the choice of priors and Markov chain Monte Carlo settings
☐	☒	For hierarchical and complex designs, identification of the appropriate level for tests and full reporting of outcomes
☐	☒	Estimates of effect sizes (e.g. Cohen's d, Pearson's r), indicating how they were calculated

Our web collection on statistics for biologists contains articles on many of the points above.

Software and code

Policy information about availability of computer code

Data collection

Clinical data were collected in REDCap (Research Electronic Data Capture) version 7.22.

Data analysis

-DIAL software version 3.0.0.12 (Alce Ingeniería, Madrid, Spain): to extract energy and nutrient intake from dietary information (table S3)
-KALUZA software version 1.2 for flow citometry analysis.
-Stata software version 16 for statistical analysis.
-GraphPad PRism v.9 for figure generation (6, S9 and S10)
-Kraken version 2.0.7-beta for amplicon data analysis
-Pavian package version 0.8.3 for OTU extraction.
-Taxonomic analysis: R software

The repository contains all the scripts and tables necessary to reproduce the results of the paper: "Fecal microbiota transplantation in HIV: A pilot, randomized, placebo-controlled study".

Bibliographic references can be found in REFERENCES.txt.

- CODE:

REFRESH_code.Rmd: code necessary to reproduce the results of the paper (Except Figure 6 and Figure S9-S9, which have been provided separately.)

REFRESH_code.Rproj: Complete R project.

nsegata-lefse-82605a2ae7b7: This code include the lefse wrapper function from 'yingstool2' github repository (https://github.com/ying14/yingtools2/wiki/Using-the-%60lefse%60-wrapper-function), a modified function that allows to use the lefse function without root access. As is specified in their github tutorial, its needed to download and unzip the folder "nsegata-lefse-82605a2ae7b7" (https://bitbucket.org/nsegata/lefse/get/default.zip) that include all the scripts necessary in order to make it work. This folder must be located in this repository if it is not.

Figure 6: This folder includes all the dataset used to generate Figure 6.

Figure S8-9: This folder includes all the dataset used to generate Figure S6.

- INPUT:

table.biom: obtained using the function kraken-biom (kraken-biom version 1.0.1, http://github.com/smdabdoub/kraken-biom) which takes all the kraken report as input.

tabla_OTUS_PAVIAN.tsv: obtained using Pavian, a web application that use all the kraken report from the samples as input and allows to download a full table that integrates all this information in a tab-separated value format.

metadata.xlsx : A four columns table (id, Donor, Randomization and abt_pre) with the metadata information necessary for the analysis.

- OUTPUT:

REFRESH_code.nb.html: HTML notebook. Report with all the code output.

REFRESH_code.html: HTML document. Report with all the code output.

VIDEO_PCA.gif: Principal Component Analysis (PCA) representing donor microbiota profiles and HIV-infected recipient microbiota community dynamics after the FMT or placebo intervention was generated using the Unweighted Unifrac distance metric.

For manuscripts utilizing custom algorithms or software that are central to the research but not yet described in published literature, software must be made available to editors and reviewers. We strongly encourage code deposition in a community repository (e.g. GitHub). See the Nature Research guidelines for submitting code & software for further information.

Data

Policy information about availability of data

All manuscripts must include a data availability statement. This statement should provide the following information, where applicable:

- Accession codes, unique identifiers, or web links for publicly available datasets
- A list of figures that have associated raw data
- A description of any restrictions on data availability

All the sequences are publicly available in the European Nucleotide Archive database under accession numbers PRJEB36786 (ERP120017). Participant’s metadata are displayed in the supplemental table. The data and custom code are available as a supplementary data file.

Field-specific reporting

Please select the one below that is the best fit for your research. If you are not sure, read the appropriate sections before making your selection.

☒ Life sciences ☐ Behavioural & social sciences ☐ Ecological, evolutionary & environmental sciences

For a reference copy of the document with all sections, see nature.com/documents/nr-reporting-summary-flat.pdf

Life sciences study design

All studies must disclose on these points even when the disclosure is negative.

Sample size	This was a pilot study, and sample size was determined based on examination of the benefit/risk ratio, resulting in a sample size of 30, after guidance by our Ethics Committee.
Data exclusions	No data exclusions
Replication	For the analysis of soluble plasma markers, samples were run in triplicate, and the means of the three replicates were used for the data analysis. For the 16S sequence experiments in feces, there were not replications, but there was a placebo control arm and longitudinal measurements.
Randomization	The study participants were randomly assigned to active or placebo in blocks of three (one per donor) by a computer-generated randomized number system (figure S2). FMT and the placebo were packaged in identically appearing capsules. The patient, the clinicians and nurses who attended the study participants, and investigators who handled patient specimens and performed the laboratory measurements, were blind to the assigned patient group. Only a third party had access to the randomization table and revealed the allocation to the investigators only

after the statistical analyses were performed.

Blinding | Investigators and patients were blinded to study allocation during data collection and statistical analyses.

Reporting for specific materials, systems and methods

We require information from authors about some types of materials, experimental systems and methods used in many studies. Here, indicate whether each material, system or method listed is relevant to your study. If you are not sure if a list item applies to your research, read the appropriate section before selecting a response.

Materials & experimental systems

n/a	Involved in the study	
☐	☒	Antibodies
☒	☐	Eukaryotic cell lines
☒	☐	Palaeontology and archaeology
☒	☐	Animals and other organisms
☐	☒	Human research participants
☐	☒	Clinical data
☒	☐	Dual use research of concern

Methods

n/a	Involved in the study	
☒	☐	ChIP-seq
☐	☒	Flow cytometry
☒	☐	MRI-based neuroimaging

Antibodies

Antibodies used | T-cell immunophenotyping from thawed PBMCs was performed with the following antibody combination: CD3-VioBlue, CD4-Fluorescein isothiocyanate (FITC), CD8-VioGreen, CD28-Phycoerythrin (PE), CD38-APC and HLA-DR-APC-Vio770, and PD-1 (PD-1-PE-Vio770).

Validation | Isotype controls were carried out.

Human research participants

Policy information about studies involving human research participants

Population characteristics | The study participants were representative of a population of middle-aged men who have sex with men with well-controlled HIV infection. Six subjects had received antibiotic treatment in the 14 weeks before the intervention, of whom three were in the FMT arm (Table S2). No significant between-groups differences were observed in food consumption and energy and nutrient intake were observed (Table S3).

Recruitment | Participants were recruited from the HIV unit of Hospital Universitario Ramón y Cajal in Madrid, Spain between January 27 and June 29, 2017. Participants were PWH on stable ART with plasma HIV RNA <37 copies/mL during at least 48 weeks and a CD4/CD8 ratio <1, as an indicator of persistent immune defects.15 Exclusion criteria were age <18 years, pregnancy, planned use of chemotherapy or antibiotics, neutropenia <500 cells/µL or CD4 counts <350 cells/µL, active infections or dysphagia. Between January and May 2017, 47 participants were screened to participate in the study; 17 were not eligible and the remaining 30 were randomized into treatment or placebo groups. A total of 29 subjects completed the 48-week follow-up (Figure S3).

Ethics oversight | Hospital Universitario Ramón y Cajal (approval number: 165/16)

Note that full information on the approval of the study protocol must also be provided in the manuscript.

Clinical data

Policy information about clinical studies

All manuscripts should comply with the ICMJEguidelines for publication of clinical research and a completedCONSORT checklist must be included with all submissions.

Clinical trial registration | Clinicaltrials.gov: NCT03008941

Study protocol | The full protocol has been attached to the submission.

Data collection | Participants were recruited from the HIV unit of Hospital Universitario Ramón y Cajal in Madrid, Spain between January and June, 2017

Outcomes | After screening for eligibility, study visits were scheduled at baseline, and at weeks 1, 2, 3, 4, 5, 6, 7, 8, 12, 24, 36 and 48. At each visit, a clinical evaluation was performed, blood and fecal samples were collected, and any adverse events were registered. A dietary assessment was performed by a nutritionist. Our primary outcomes were safety and tolerability. Our secondary outcomes were changes in CD4+ and CD8+ T cells and CD4/CD8 ratio from baseline to week 48. Gastrointestinal tolerance was assessed using a questionnaire and scoring the severity of gastrointestinal symptoms on a 4-point scale and stool characteristics using the Bristol stool chart. We measured variations in the microbiota composition (alpha and beta diversity metrics) and engraftment of donor's microbiota on recipients (Unifrac distances from recipient samples to donor's microbiota).

We analyzed changes in systemic biomarkers based on their independent predictive value of mortality or their relevance in HIV immunopathogenesis24,25. Exploratory markers included changes in the plasma markers of inflammation (C Reactive Protein [CRP], soluble CD163 [sCD163], soluble tumor necrosis factor receptor II [sTNFr-II], monocyte/macrophage activation (soluble CD14 [sCD14], interferon-gamma induced protein 10 [IP-10]), bacterial translocation (lipoteichoic acid [LTA], lipopolysaccharide-binding protein [LBP]), coagulation (D-dimers), T cell activation markers (% of HLADR+CD38+ T-cells), T-cell senescence markers (% of CD28- T-cells), and T-cell exhaustion markers (%PD-1+ of T-cells).

Flow Cytometry

Plots

Confirm that:

- [x] The axis labels state the marker and fluorochrome used (e.g. CD4-FITC).
- [x] The axis scales are clearly visible. Include numbers along axes only for bottom left plot of group (a 'group' is an analysis of identical markers).
- [x] All plots are contour plots with outliers or pseudocolor plots.
- [x] A numerical value for number of cells or percentage (with statistics) is provided.

Methodology

Sample preparation	For T-cell immunophenotyping, peripheral blood mononuclear cells (PBMCs) were isolated by Ficoll-Hypaque gradient-centrifugation and immediately stored in liquid nitrogen. T-cell immunophenotyping from thawed PBMCs was performed with the following antibody combination: CD3-VioBlue, CD4-Fluorescein isothiocyanate (FITC), CD8-VioGreen, CD28-Phycoerythrin (PE), CD38-APC and HLA-DR-APC-Vio770, and PD-1 (PD-1-PE-Vio770). Antibodies were purchased from Myltenyi Biotec (Bergisch Gladbach, Germany), and isotype controls were carried out. Briefly, PBMCs were incubated with the antibodies for 20 min at 4°C, washed and resuspended in PBS containing 1% azide. Cells were analyzed using a Gallios flow cytometer (Beckman-Coulter, CA, USA). After initially gating lymphocytes according to morphological parameters, at least 30000 CD3+ T-cells were gated for each sample and analyzed with Kaluza software (Beckman-Coulter).
Instrument	Cells were analyzed using a Gallios flow cytometer (Beckman-Coulter, CA, USA).
Software	Kaluza software (Beckman-Coulter).
Cell population abundance	At least 30000 CD3+ T-cells were collected for each sample
Gating strategy	After initially gating lymphocytes according to morphological parameters only singlets were further analyzed. Then, cells with CD3+ phenotype were analyzed using CD4 and CD8 antibodies. Activated T cells were defined as the coexpression of CD38 and HLA-DR antibodies. Also, CD28- cells and PD-1+ cells were analysed in the CD4+ or CD8+ cell subpopulation.

- [x] Tick this box to confirm that a figure exemplifying the gating strategy is provided in the Supplementary Information.
